# Quantitative Genetics and Genomics Converge to Accelerate Forest Tree Breeding

**DOI:** 10.3389/fpls.2018.01693

**Published:** 2018-11-22

**Authors:** Dario Grattapaglia, Orzenil B. Silva-Junior, Rafael T. Resende, Eduardo P. Cappa, Bárbara S. F. Müller, Biyue Tan, Fikret Isik, Blaise Ratcliffe, Yousry A. El-Kassaby

**Affiliations:** ^1^EMBRAPA Recursos Genéticos e Biotecnologia Brasília, Brazil; ^2^Programa de Ciências Genômicas e Biotecnologia Universidade Católica de Brasília, Brasília, Brazil; ^3^Departamento de Biologia Celular Universidade de Brasília, Brasília, Brazil; ^4^Department of Forestry and Environmental Resources, North Carolina State University Raleigh, NC, United States; ^5^Centro de Investigación de Recursos Naturales, Instituto de Recursos Biológicos INTA, Buenos Aires, Argentina; ^6^Consejo Nacional de Investigaciones Científicas y Técnicas Buenos Aires, Argentina; ^7^Biomaterials Division Stora Enso AB, Stockholm, Sweden; ^8^Department of Forest and Conservation Sciences, Faculty of Forestry, University of British Columbia Vancouver, BC, Canada

**Keywords:** genomic selection (GS), tree breeding, quantitative genetics, whole-genome regression, single nucleotide polymorphisms (SNP), marker assisted selection (MAS), realized genomic relationship

## Abstract

Forest tree breeding has been successful at delivering genetically improved material for multiple traits based on recurrent cycles of selection, mating, and testing. However, long breeding cycles, late flowering, variable juvenile-mature correlations, emerging pests and diseases, climate, and market changes, all pose formidable challenges. Genetic dissection approaches such as quantitative trait mapping and association genetics have been fruitless to effectively drive operational marker-assisted selection (MAS) in forest trees, largely because of the complex multifactorial inheritance of most, if not all traits of interest. The convergence of high-throughput genomics and quantitative genetics has established two new paradigms that are changing contemporary tree breeding dogmas. Genomic selection (GS) uses large number of genome-wide markers to predict complex phenotypes. It has the potential to accelerate breeding cycles, increase selection intensity and improve the accuracy of breeding values. Realized genomic relationships matrices, on the other hand, provide innovations in genetic parameters' estimation and breeding approaches by tracking the variation arising from random Mendelian segregation in pedigrees. In light of a recent flow of promising experimental results, here we briefly review the main concepts, analytical tools and remaining challenges that currently underlie the application of genomics data to tree breeding. With easy and cost-effective genotyping, we are now at the brink of extensive adoption of GS in tree breeding. Areas for future GS research include optimizing strategies for updating prediction models, adding validated functional genomics data to improve prediction accuracy, and integrating genomic and multi-environment data for forecasting the performance of genetic material in untested sites or under changing climate scenarios. The buildup of phenotypic and genome-wide data across large-scale breeding populations and advances in computational prediction of discrete genomic features should also provide opportunities to enhance the application of genomics to tree breeding.

## Introduction

Forest tree breeding encompasses a number of steps to increase the frequency of advantageous alleles for several traits concurrently in a target population. Recurrent cycles of selection ultimately result in genetically improved planting material by maximizing genetic gain per unit time in the most cost-effective way (Namkoong et al., 1988; White et al., 2007). Long breeding cycles, late and poor flowering, weak juvenile-mature correlations and changes in climate, market demands and emerging pest and disease pressures, pose, however, daunting challenges. The advancement and ultimate output of tree breeding programs are therefore highly conditional on the length of a breeding cycle. To maximize genetic gains per unit time, extensive efforts in tree breeding were devoted to the two fundamental means by which the length of a breeding cycle can be decreased, namely, early selection and accelerated breeding. While the former is based on the understanding of juvenile-mature correlations and the practice of selection on juvenile traits (Williams, 1988), the latter involved early flower induction methods with hormone, stress treatments, and top grafting (Greenwood et al., 1991; Hasan and Reid, 1995). In the late 80s, the advent of DNA markers and two seminal papers on the dissection of discrete Mendelian factors underlying quantitative traits (Lander and Botstein, 1989), and marker-assisted selection (MAS) (Lande and Thompson, 1990), were seen as powerful tools to overcome the time challenge of tree breeding (El-Kassaby, 1982; Neale and Williams, 1991; Grattapaglia et al., 1992; Williams and Neale, 1992).

Here we cover the current state of the science on the general theme of optimizing and accelerating tree breeding using genomic technologies. A brief overview of the path across QTL (quantitative trait loci) and association mapping is first presented. It provides a quick historical perspective on how and why we reached the point of convergence between quantitative genetics and genomics. Furthermore, it also serves to substantiate the fact that reductionist “genetic dissection” approaches or attempts to use single candidate genes or diffuse, indirect information from transcriptomics, have not proven useful for breeding practice and are therefore not discussed further. We focus on the factors that affect and the challenges that remain to fully integrate genomic data in tree breeding in light of the recent promising results of whole-genome prediction. Although making predictions is difficult “especially about the future” as Niels Bohr and others once amusingly said, we attempt to look at the near future of tree breeding when genotyping, whole genome sequencing and computational prediction of genomic features for thousands of trees will not be limiting. We anticipate a future where the progressive advances made possible by routine genomic selection (GS) in multiple large populations will provide a more powerful platform to revisit the discovery of discrete genomic elements that may further enhance whole-genome phenotype prediction and eventually allow direct discrete interventions at the DNA sequence level.

## The path from genetic dissection to genomic selection

The prospects of MAS for forest trees was properly doubted early on, limiting its potential value to specific genetic backgrounds resulting from linkage equilibrium of forest tree populations, QTLs interacting with environments and changes of allele frequencies across generations (Strauss et al., 1992). Notwithstanding those sound advices, a number of QTL mapping experiments in the major conifers and eucalypts advanced, encouraged by the promising results of QTL mapping in inbred crops and model systems. In retrospect it is startling to consider how far removed from real-life tree breeding those bi-parental QTL mapping studies in forest trees were (Grattapaglia, 2017). The motivating hypothesis was that it would be possible to locate and estimate the effects of most individual QTLs underlying complex traits in every population and environment and implement them in tree breeding practice. A substantial number of studies reporting hundreds QTLs in forest trees was reported (reviewed in (Kirst et al., 2004; Grattapaglia et al., 2009; Neale and Kremer, 2011). Although several supposedly “major effect” QTLs were found in those early studies, those proved to be largely overestimated in effect size and underestimated in number. Indeed, subsequent multi-family experiments and larger sample sizes, revealed significantly larger numbers of QTLs with correspondingly smaller effects and inconsistent performance across environments and genetic backgrounds (Ukrainetz et al., 2008; Novaes et al., 2009; Thumma et al., 2010; Gion et al., 2011).

To solve the perceived shortcomings of QTL detection in single mapping families, association genetics was put forward as a way to provide population-wide marker–trait associations applicable to breeding (Neale and Savolainen, 2004). The limitation of methods to interrogate DNA polymorphisms at the time only allowed candidate-gene approaches (Thumma et al., 2005; Gonzalez-Martinez et al., 2007), which were then followed by genome-wide association mapping (GWAS) in several forest tree species (Beaulieu et al., 2011; Cumbie et al., 2011; Cappa et al., 2013; Porth et al., 2013; Mckown et al., 2014). However, irrespective of the marker density used, population size and improved analytical methods to account for low-frequency variants (Fahrenkrog et al., 2017; Müller et al., 2017, 2018; Resende et al., 2017a), only few polymorphisms of very modest effect have been detected, largely still lacking independent validation, the cornerstone for the scientific credibility of GWAS results. In effect, after 25 years of research efforts based on the principle and experimental approaches of genetic dissection of quantitative traits, no translation of such efforts to operational tree breeding was achieved (Grattapaglia et al., 2009; Grattapaglia, 2014; Isik, 2014).

The ineffectiveness of fully dissecting complex traits, and the limitations of MAS has not been exclusive to forest trees, but has also been recognized in crops (Bernardo, 2008) and domestic animals (Dekkers, 2004). This realization has caused a significant shift in the paradigm and technical approach to plant and animal MAS. These fields have now moved from the *a priori* discovery of discrete marker-trait associations to the capture of the whole-genome effect assisted by DNA marker data, harmonizing with the multifactorial polygenic nature of quantitative genetics, as predicted by Fisher's infinitesimal model (Fisher, 1918). This shift was only possible following the development of improved and accessible genomic technologies that allow interrogating thousands of genome-wide single-nucleotide polymorphisms (SNPs) using cost effective platforms. The concept of using the “total allelic” (Nejati-Javaremi et al., 1997) or “total genomic” (Haley and Visscher, 1998) relationship from marker data to derive estimates of breeding values was later termed “Genomic Selection” (GS) in Meuwissen et al. (2001) seminal paper. It demonstrated that “*the selection on genetic values predicted from markers could substantially increase the rate of genetic gain per unit time in animals and plants, especially if combined with techniques to shorten the generation interval*.”

**Figure 1:**
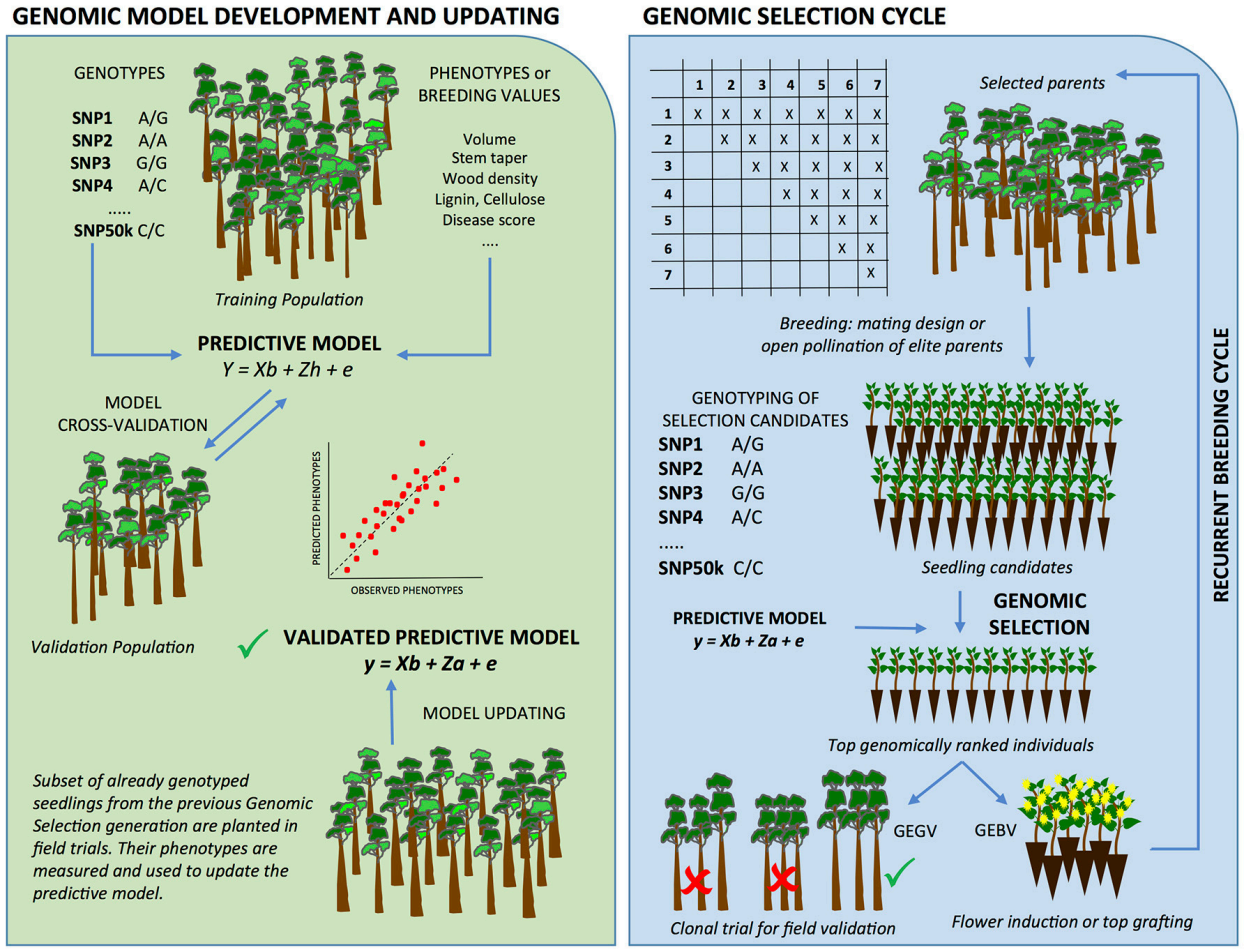
Genomic selection in forest trees. GS begins with the development of a predictive model for the traits of interest **(Left panel)**, which are then used in the GS cycles **(Right panel)** and progressively updated. GS uses genome-wide markers whose effects on the phenotype are estimated concurrently in a large and representative “training population” of individuals without applying severe significance tests. Markers are retained as forecasters of phenotypes in prediction models to be later applied to “selection candidates” for which only genotypes are collected. The prediction models are cross-validated against a “validation population,” a set of individuals of the same reference population that were not used for the estimation of marker effects. Once a prediction model is shown to provide adequate accuracy, it can be used in the GS cycle. An array of selection candidates - full of half-sib families derived from crossing either the original elite parents of the training set, or elite individuals selected in the training set - are genotyped and have their breeding values (GEBV) and/or genotypic values (GEGV; additive + non-additive effects) estimated using the model developed earlier. Top ranked seedlings for GEBV are subject to early flower induction and inter-mated to create the next generation of breeding. Top ranked seedlings for GEGV are clonally propagated and tested in verification clonal trials where elite clones are eventually selected for operational plantation. Additionally, all or subsets of the already genotyped selection candidates are planted in experimental design and phenotyped at the target selection age to provide genotype and trait data for GS model updating as GS generations advance and climate changes.

GS employs a genome-wide panel of markers, typically SNP (single nucleotide polymorphism), whose effects on the phenotype are estimated in a “training” population. In forest trees, such a training set is usually composed by sampling one to a few thousand individuals in progeny trials derived from mating a few dozen parents that constitute the target elite germplasm bred. SNPs are used to build prediction models to be later applied to “selection candidates” for which only genotypes are gathered and phenotypes are predicted by the genomic data. The prediction models are cross-validated against a “validation” population, a set of genetically related individuals to the training set but that did not participate in the estimation of marker effects. A prediction model that delivers a high correlation between the observed and predicted breeding values is subsequently used in the breeding phase to calculate the genomic estimated breeding or total genotypic values of the selection candidates (Figure 1). GS fundamentally exploits the genetic relationship between the training population and the prospective selection candidates and to a lesser extent the linkage disequilibrium (LD) between marker data and QTL effects. By precluding prior discrete marker selection derived from rigorous significance tests, and by estimating marker effects in a larger and breeding-representative population of trees, GS captures substantial proportions of the heritability contributed by the large numbers of genomic effects that QTL mapping or GWAS are, on principle, neither able nor intended to capture.

## Perspectives of genomic selection in tree breeding

GS can have a substantial impact on the rate of genetic gain. Let's recall Falconer's breeder's equation (ΔG = *ir*σ_*A*_/*L*) (Falconer, 1989), where *i* is the selection intensity; *r* is the accuracy of selection, or heritability in the original Falconer's expression, corresponding to the correlation between the estimated and true breeding values; σ_*A*_ is the additive-genetic standard deviation of the trait under inspection; and *L* is the generation interval. GS can increase the rate of genetic gain of breeding cycle by increasing (*i*) because the phenotypes of a much larger number of seedlings in the nursery can be predicted with marker data compared to the number of trees that can be tested in conventional field progeny trials. Additionally, the use of realized genomic relationships is associated with increased accuracy in estimating σ_*A*_ and breeding values *(r)* (Hayes et al., 2009; El-Dien et al., 2018). Yet, in forest trees, the potentially greatest impact of GS on the rate of genetic progress will originate from decreasing (*L*). Phenotypes of the selection candidates can be predicted at very early ages, for example, when the seedlings are a few weeks old. GS not only could preclude or at least enhance the efficiency of progeny testing but would also optimize clonal testing phases by advancing a smaller number of pre-selected trees to be assessed in multi-site expanded clonal trials (Resende et al., 2012a) (Figure 1). In conifers, GS coupled to somatic embryogenesis for clonal propagation of elite genotypes could allow selecting elite zygotic embryos based on their genomic value saving a significant amount of time, and avoiding the costs and uncertainties currently involved in cryopreservation rescue (Resende et al., 2012b). Additionally, GS will allow simultaneous and early selection for multiple trait in large numbers of individuals, an impossible task in conventional tree breeding that currently largely adopts tandem selection. The final impact of GS would therefore be a significant improvement in the general efficiency of a tree breeding program, provided, of course, that genotyping is inexpensive and GS models are accurate.

What makes GS distinctive from what tree breeders have done so far is that instead of relying uniquely on the expected pedigree, frequently prone to errors, DNA data allows one to build additive and non-additive genomic relationship matrices that more accurately specify the relationships among individuals and simultaneously account for contemporary as well as historical pedigree. This procedure not only allows rectifying pedigree inaccuracies, but critically it captures the within family variation resulting from random Mendelian segregation term. Accordingly, the realized genetic covariances are now based on the actual fraction of the genome that is identical by descent or by state between individuals (Vanraden, 2008). It has been shown in a number of studies in forest trees that realized genomic relationships can produce more accurate predictions than pedigrees alone (Munoz et al., 2014; El-Dien et al., 2015, 2018; Bouvet et al., 2016; Cappa et al., 2017, 2018; Tan et al., 2018). Additionally, the realized genomic relationships of a small subset of the progeny testing population have been effectively combined with a substantially large proportion of un-genotyped individuals in a single-step analysis (Legarra et al., 2009). This method was dubbed “HBLUP,” since the best linear unbiased predictors (BLUPs) of breeding values are derived using a single (***H***) genetic covariance matrix that combines the pedigree-based average numerator relationship matrix (***A***) with the marker-based relationship matrix (***G***). HBLUP increases the precision of the genetic parameters generated from traditional pedigrees as shown in recent studies with forest trees (Cappa et al., 2017, 2018; Ratcliffe et al., 2017).

## GS: advances and challenges in forest trees

A comprehensive and time-lined list of empirical GS reports in forest tree species was recently published (Grattapaglia, 2017) and it is now updated in Table 1. Prediction accuracies have been largely very good, matching or surpassing those obtainable by pedigree-based phenotypic selection, in line with former simulations (Grattapaglia and Resende, 2011; Iwata et al., 2011; Denis and Bouvet, 2013). When considering the practicalities of tree breeding, however, a number of factors that affect the prospects of GS have to be considered, including the composition of training populations, analytical methods, genotype x environment interaction (G^*^E), age-age correlations, the long term models performance and cost and quality of DNA marker data. All these have been the subject of research and reviewed in detail in the context of tree breeding by Grattapaglia (2017) and are briefly discussed below in light of the experimental results reported to date in forest trees.

**Table 1:** Timeline summary of experimental genomic selection studies in forest tree species published to date.

**Forest tree**	**DNA marker data**	**Traits analyzed**	**References**
Eucalypts (*Eucalyptus grandis x E. urophylla, E. camaldulensis*- hybrids)	3,129 DArT array	Growth and wood quality	Grattapaglia et al., 2011 Resende et al., 2012a
Loblolly pine (*Pinus taeda*)	4,852 SNP chip	Growth, wood and disease	Resende et al., 2012b Resende et al., 2012c Munoz et al., 2014 De Almeida et al., 2016
Loblolly pine (*Pinus taeda*)	3,461 SNP chip	Growth and wood quality	Zapata-Valenzuela et al., 2012 Zapata-Valenzuela et al., 2013
Eucalypts (*Eucalyptus grandis x E. urophylla*- hybrids)	29,090 SNP EuCHIP60K	Growth and wood quality	Lima, 2014
White spruce (*Picea glauca*)	6,932 SNP chip	Growth and wood quality	Beaulieu et al., 2014a Beaulieu et al., 2014b Ratcliffe et al., 2017
Interior spruce (*Picea glauca x P. engelmannii*)	8,868 to 62,198 GbS SNPs	Growth and wood quality	El-Dien et al., 2015 Ratcliffe et al., 2015 El-Dien et al., 2018
Maritime pine (*Pinus pinaster*)	4,332 SNP chip	Growth and form	Isik et al., 2016 Bartholome et al., 2016
Eucalypts (*Eucalyptus grandis x E. urophylla*- hybrids)	3,303 DArTseq SNPs	Growth	Bouvet et al., 2016
Eucalypt (*Eucalyptus pellita* and *E. benthamii*)	19,506 SNP EuCHIP60K	Growth	Müller et al., 2017
Eucalypt (*Eucalyptus grandis x E. urophylla*- hybrids)	24,806 SNP EuCHIP60K	Growth and wood quality	Resende et al., 2017b
Eucalypt (*Eucalyptus globulus*)	12,000 SNP EuCHIP60K	Growth and wood quality	Duran et al., 2017
Black spruce (*Picea mariana*)	4,993 SNP chip	Growth and wood quality	Lenz et al., 2017
Eucalypt (*Eucalyptus grandis*)	2,816 DArT array	Growth	Cappa et al., 2017 Cappa et al., 2018
Eucalypt (*Eucalyptus grandis x E. urophylla*- hybrids)	41,304 SNP EuCHIP60K	Growth and wood quality	Tan et al., 2017 Tan et al., 2018
Douglas fir (*Pseudotsuga menziesii*)	69,551 exome capture SNPs	Growth and wood quality	Thistlethwaite et al., 2017
Norway spruce (*Picea abies*)	116,765 exome capture SNPs	Growth and wood quality	Chen et al., 2018
Eucalypt (*Eucalyptus nitens*)	12,236 SNP EuCHIP60K	Growth and wood quality	Suontama et al., 2018
Eucalypt (*Eucalyptus polybractea*)	Shallow whole genome sequencing; up to 500,000 SNPs	Foliar terpene yield traits	Kainer et al., 2018
Eucalypt (*Eucalyptus grandis x E. urophylla*- hybrids)	40,932 SNP EuCHIP60K and 55,772 capture probe SNP	Growth and wood quality	De Moraes et al., 2018

*Studies that investigated different aspects of GS but used partially or totally the same breeding population data (genotypes and/or phenotypes) are listed in the same entry*.

GS experiments in forest trees have capitalized on the existing structure and diversity of breeding populations and their designs that account for the expected relationship between training and prospective selection candidates. Training populations of several hundred to a few thousand individuals sampled from existing progeny trials with effective population sizes consistent with those used in operational breeding have provided good predictions in essentially all studies and for all traits. Analytical methods differing with respect to the presumed trait architecture have been used and compared. In all studies, the ridge regression best linear unbiased prediction (RR-BLUP), with marker effects treated as random, normally distributed with common variance, has been very efficient. RR-BLUP has been equivalent to Genomic BLUP (GBLUP), providing the best conciliation between prediction efficiency and fast computation, while also revealing that essentially all major traits in forest trees fit the infinitesimal model (Resende et al., 2012c; Beaulieu et al., 2014b; Ratcliffe et al., 2015; Isik et al., 2016; Müller et al., 2017; Tan et al., 2017; Chen et al., 2018). Still, additional research in this area is warranted especially as prior functional information on genomic regions of slightly larger effect might emerge, for example, for disease resistance traits as shown for prediction of fusiform rust resistance in loblolly pine (Resende et al., 2012c).

Ever since the first experimental GS studies in forest trees (Grattapaglia et al., 2011; Resende et al., 2012a,b), it became clear that prediction accuracies are mainly driven by genetic relationship between training and validation sets and are dependent on G^*^E and age-age correlations. Predictions will be most effective at the same age and in the same environment where the prediction model was trained. Further studies in conifers (Zapata-Valenzuela et al., 2013; Beaulieu et al., 2014a,b; El-Dien et al., 2015; Ratcliffe et al., 2015; Thistlethwaite et al., 2017; Chen et al., 2018), and eucalypts (Müller et al., 2017; Tan et al., 2017), corroborated the key significance of genetic relationships and the impact of G^*^E and age-age correlations, consistent with findings in domestic animals and crop plants (Lin et al., 2014; Van Eenennaam et al., 2014). While data from G^*^E or age-age correlation studies will shed light on what to expect from genomic prediction, assuring that the target environment of future selection candidates will be equivalent to the one where models were originally trained is a challenging issue for GS (Heslot et al., 2015). Regular retraining of GS models by incorporating phenotypes collected in breeding generations closer to the current (Iwata et al., 2011) are expected to mitigate this problem, and will be especially essential in light of climate fluctuations. Research efforts in this area are highly needed and will come as GS programs advance, coupled to innovations in phenotyping platforms that integrate remote sensing, spatial and geographic information systems (Dungey et al., 2018).

Notwithstanding the encouraging estimates of predictive ability, most studies in forest trees used contemporary training and validation sets and thus have not yet been able to adequately assess the realized performance of GS across generations at a larger scale, but results on this topic are imminent. However, given that the relationship between parents and progeny are accurately captured by DNA marker data, and environments should be relatively stable across close generations, it is expected that the performance will be equivalent to current estimates in contemporary sets. In *Pinus pinaster*, preliminary promising results of inter-generation prediction were reported by training models with parents and progeny in the same set (Isik et al., 2016), and later using parents and grandparents to predict in the subsequent generation, albeit with limited effective population sizes (Bartholome et al., 2016). However, this outcome was not observed in a three-generation study of *Pseudotsuga menziesii* (El-Kassaby, personal communication). Model updating strategies will therefore be crucial to counteract the decay of relatedness and LD between the original training set and selection candidates as generations of breeding advance, as shown by simulations for eucalypt breeding (Denis and Bouvet, 2013).

In the past 2 years, a number of additional experimental GS studies have been reported (Cappa et al., 2017, 2018; Duran et al., 2017; Lenz et al., 2017; Müller et al., 2017; Ratcliffe et al., 2017; Tan et al., 2017, 2018; Thistlethwaite et al., 2017) (Table 1; Resende et al., 2017b; Chen et al., 2018; De Moraes et al., 2018; El-Dien et al., 2018; Kainer et al., 2018; Suontama et al., 2018). Many of them in species of *Eucalyptus* for which public high-throughput genotyping platforms of DArT (Sansaloni et al., 2010) and SNPs (Silva-Junior et al., 2015) have been available. Access to such resources for eucalypts also allowed improved precision of genetic parameter estimates, pedigree reconstruction and inbreeding studies (Telfer et al., 2015; Klápště et al., 2017; Müller et al., 2017). This clearly points to the fact that the advancement of research and operational adoption of genomics into breeding is strongly dependent on the availability of public, robust, cost-accessible and portable SNP genotyping platforms. The success of GS or any other genomic-based breeding approach will rely on high data quality, as one has to be able to genotype SNPs across generations with high reproducibility and negligible missing data. Although shallow whole genome sequencing (Kainer et al., 2018), genotyping-by-sequencing (GbS) (El-Dien et al., 2015) and sequence capture (Thistlethwaite et al., 2017; Chen et al., 2018; De Moraes et al., 2018) have also been used for GS in trees, currently fixed SNP arrays provide the gold standard of data reproducibility across samples batches and laboratories. Additionally, SNP array data are breeder friendly, available from multiple service providers, easily manageable and stored without the cost and logistics of sequence data transfer, storage and analysis. This and a significant recent drop in array costs, making them as cost-effective as sequence-based methods, has motivated a large international effort to develop SNP arrays for all main planted conifers (F. Isik pers. comm.), and a second generation, higher density optimized SNP array for species of *Eucalyptus* and *Corymbia* (O.B. Silva-Junior and D. Grattapaglia pers. comm.). The use of a common SNP genotyping array across breeding programs of different organizations will be a key issue to provide the necessary economy of scale to integrate genomics into breeding.

## A look to the near future

With easy access to SNP genotyping and positive results in essentially all major forest trees, we are now at the brink of widespread adoption of genomic prediction data, thus realizing the early promises of MAS in forest tree breeding. In addition to the outstanding research challenges discussed above, a promising area to enhance the value of genomic data will involve the inclusion of environmental co-variables in GS models as already shown in crops (Jarquin et al., 2014; Saint Pierre et al., 2016). The integration of multi-environment trials data will be strategic for predicting performance in unobserved environments, identifying suitable sites for evaluating or deploying genetic material and predicting climate change scenarios. While predicting the performance of untested clones or families can be accurate when there is knowledge of genomic relatedness, correspondingly, the performance in yet unobserved or future environments could be forecasted if there is data about those environments as shown for recommendation of *Eucalyptus* clones (Marcatti et al., 2017). Resources such as ClimateNA (Wang et al., 2016) and the NASA POWER project (Stackhouse, 2014) offer multitudes of historical and predicted future environmental data. Because environmental variables that define the correlation between growing conditions are trait specific, research on those most appropriate for inclusion in genomic prediction models will be essential.

Another area that will demand research comes from the evolution of sequencing technologies in moving from sparse SNP data to sequence data for GS. Apart from the challenge and cost of managing massive next generation sequencing data sets for large numbers of individuals in a breeding program framework, in theory, if sequence data were used instead of dense SNPs, accuracy should increase because rare causal alleles would be better captured in predictive models. Until now, however, simulation and experimental studies in domestic animals have shown that whole-genome sequence data does not increase accuracy when LD has a slow decay pattern (Macleod et al., 2014; Forneris et al., 2017; Vanraden et al., 2017), unless very precise prior estimates on the functionality of particular SNPs exist (Perez-Enciso et al., 2015). Increasing the availability and quality of functional data on specific genomic regions might therefore, be warranted.

The success of whole-genome prediction and the poor outcome of dissection approaches in identifying functional quantitative trait nucleotides, have contributed nevertheless to an exciting new perspective for the study of complex trait variation. A clear pattern has emerged in annual plants indicating that the association signal of common variants in large sample sizes, although spread across the entire genome, is heavily concentrated in regulatory DNA in open chromatin marked by deoxyribonuclease hypersensitive sites (Sullivan et al., 2014; Rodgers-Melnick et al., 2016; Swinnen et al., 2016). In these plants, cis-regulatory elements (CREs) associated with open chromatin such as promoters and enhancers regulating gene expression may contain close to half of all variants influencing traits. As GS implementation advances and large datasets of several thousand trees across unrelated populations are collected, opportunities will emerge for joint and meta-GWAS, as recently described in *Eucalyptus* (Müller et al., 2018). At the same time, chromatin accessibility and gene network data as reported for *Eucalyptus* (Hussey et al., 2017) and *Populus* (Zinkgraf et al., 2017) will become increasingly available which, combined with data from highly powered SNP-trait association studies, should provide new avenues for computational predictive discovery of key regulatory elements in the genome. The progress of such integrative approaches based on large genotype and phenotype datasets might, thus, result in additional clues toward understanding the complex connections and interactions between discrete genomic elements and continuous phenotypic trait variation, ultimately enhancing tree breeding practice.

## Author contributions

DG drafted the first version of the manuscript and all co-authors subsequently contributed to it by editing and formatting the final version.

### Conflict of interest statement

The authors declare that the research was conducted in the absence of any commercial or financial relationships that could be construed as a potential conflict of interest.
